# The Influence of Environmental Values on Consumer Intentions to Participate in Agritourism—A Model to Extend TPB

**DOI:** 10.1007/s10806-022-09881-8

**Published:** 2022-08-08

**Authors:** Zinan Zhao, Yongji Xue, Lili Geng, Ye Xu, Nyingone Ndongo Meline

**Affiliations:** 1 School of Economics and Management, Beijing Forestry University, 100083 Beijing, China; 2 College of Business, Concordia University Chicago, IL60305 River Forest, USA

**Keywords:** Environmental values, Consumer intentions, Agritourism, Theory of planned behaviour

## Abstract

This study examines the influence of environmental values on consumer intentions to participate in agritourism through the theory of planned behaviour (TPB) and value-belief-norm (VBN) theory. It proposes an integrative model by adding two variables, i.e., environmental benefits and the human-nature coordination concept, to the TPB. The study employs a questionnaire survey method and a sample of 640, which was statistically analysed through structural equation modeling (SEM). The results reveal that the “environmental values-attitudes-behavioural intentions” framework has scientific applicability in agritourism. Environmental values, measured through the variables environmental benefits and the human-nature coordination concept, are positively correlated directly or indirectly with agritourism consumption intentions, while attitudes and subjective norms serve as mediators. However, the mediating effect of perceived behavioural control is not statistically significant, indicating making efforts to influence attitudes and subjective norms is more useful for and effective in stimulating the public’s intentions towards agritourism. As this study tests the hypotheses with empirical data, it provides practical implications for policy-makers and programme managers.

## Introduction

Over the past three decades, the Chinese government has prioritized sustainable rural development and poverty alleviation. At the 19th Session of the National Congress of the Communist Party of China in 2017, the Chinese government announced its intention to accelerate rural revitalization (The 19th CCCPC, 2017). One significant approach for rural revitalization combines industrial development with agricultural supply-side structural reform (Xingping, 2019). Specifically, the approach aims to strengthen industrial agricultural supply chains, promote agricultural efficiency, increase farmers’ income, and preserve rural beauty and prosperity through agritourism development and other green agricultural development models (Huang et al., 2014). Under the policy of promoting rural revitalization, agritourism, which is a new agricultural development pattern, has emerged rapidly in most rural areas in China. Between 2010 and 2019, the total registered number of agritourism-related enterprises increased from 26,000 to 216,000 (Lv, 2020). In 2019, 3.2 billion tourists participated in agritourism, generating an operating revenue of more than 850 billion yuan (Lv, 2020).

Since 2010, China’s tourism industry has experienced a high growth trend; according to research and China’s Investment Strategy Report, this trend is also reflected in agritourism (Huang et al., 2014). Agritourism first developed from the simple practice of visiting agricultural farms for sightseeing and then grew to include vacation experience tourism in the 1980s (Wang and Zhang, 2016). Agritourism is mainly characterized by exploring agricultural production, processing, and sales, where customers experience folkways and enjoyable feelings from agricultural activities (Xingping, 2019). Not only does agritourism have the potential to meet people’s needs for leisure and enhance their knowledge and understanding of the agricultural environment, but it can also help develop agricultural resources, improve the local infrastructure, and increase income in rural areas.

China’s economic reform and opening-up has led to gradual social structural change, including growing urbanization and a shrinking rural sector. Demographers estimate that in the past four decades, over 640 million farmers departed from their hometowns and moved to a Chinese city (Dan, 2019). In 1978, only 17.9% (170 million) of the Chinese population lived in cities, but by 2018, this number had grown to approximately 58.5% (810 million) (Lu et al., 2019). However, after a period of fast-paced and high-pressure urban life, leisurely and comfortable rural areas have become attractive to urban residents as places to rest and relax. The rising Chinese urban middle class has created a large market for agritourism (Xue et al., 2021).

Agritourism is also considered a global sunrise industry (Chiodo et al., 2019). Rambodagedara, Silva, and Perera (2015) point out that agritourism has become increasingly popular around the world because it provides farmers with a specific focus for diversified income-generating activities that ultimately lead to rural development. Currently, agritourism creates an alternative source of income for rural communities and has become a mature practice in many developed countries. It is being practised in many countries, such as Thailand, Malaysia, India, Nepal, Bhutan, and Indonesia, providing numerous benefits to rural communities (Gurrieri et al., 2014). Finally, there will likely be a tourism boom after the coronavirus pandemic is over. Since the outbreak of COVID-19 at the beginning of 2020, many affected countries and cities have adopted isolation responses to restrict travel (Harari, 2020). It is projected that many people will seek to participate in tourism when the outbreak is over.

Increasing numbers of people have a strong intention to participate in agritourism because the rural environment brings them physical and mental relaxation. However, agritourism places more stress on the natural environment. Deteriorating rural environments are likely to reduce consumers’ intentions towards agritourism. In the current era, people tend to pursue a leisurely, comfortable, and convenient tourism experience. Persuading people to protect the environment through normative measures is likely to cause social confrontation and contradictions, which are an obstacle to the cultivation of green tourism. Therefore, it is particularly important to explore the subjective values guiding consumers’ intentions towards agritourism at the individual micro level (He et al., 2019). Tourists’ behavioural intentions to protect the environment are connected to the promotion of sustainable development, which is dependent upon their subjective orientation towards consuming environmentally friendly tourism activities, such as agritourism. Thus, environmental values are the internal driving force of this behavioural intention (Pan & Wang, 2018). Therefore, it is necessary and meaningful to study the impact of environmental values on consumer intentions and behaviours towards agritourism.

Ajzen’s (1991) theory of planned behaviour (TPB), which was based on Ajzen and Fishbein’s (1977) theory of reasoned action (TRA), has become one of the most popular and influential theoretical frameworks for studying human intentions and behaviours (Ajzen, 1991). The TPB has been widely applied in various industries and fields and to conduct research in different disciplines. However, existing research on consumer intentions towards agritourism lacks empirical data and quantitative analysis to determine the interrelationship between values, intentions, and behaviours. This study utilizes relevant questionnaire data and applies the TPB to agritourism. Our results extend the TPB model by incorporating environmental values. Existing research on the TPB demonstrates that attitudes, subjective norms, and perceived behavioural control are three significant variables that positively correlate with people’s behavioural intentions, while behavioural intentions directly impact behaviour (Cheng et al., 2011).

This article introduces two factors pertaining to environmental values, i.e., environmental benefits and the human-nature coordination concept, which are used to explore the internal mechanisms between environmental values and consumer intentions towards agritourism. The relationship between these two internal factors is determined through structural equation modeling (SEM), which provides the path coefficient of the interaction mechanisms. The results have significant policy implications for the government, as they reveal the important underlying mechanisms that influence people’s orientation towards and, thus, the development of agritourism.

The article contains three sections: the literature review, the methodology section, and the discussion. The first section reviews the related literature on environmental values and consumer intentions towards agritourism and states the hypotheses. The methodology section includes a description of the research methods, sample collection, research process, scale design, data analysis, and results inspection. The last section further discusses the results, draws conclusions, and gives practical suggestions concerning agritourism and concludes with directions for future research.

## Literature Review and Hypotheses

### Theory of Planned Behaviour (TPB) and Value-Belief-Norm (VBN) Theory

The term “values” has several varying definitions and has been used in vastly different ways in a variety of disciplines and contexts (Brown, 1984). The definition of “values” used in this article is ‘the principles and beliefs that influence the behavior and way of life of a particular group or community’ (Millard, 1949; Tadaki et al., 2017). In terms of environmental values, Linda Steg (2016) clarified that environmental values steer people’s views on the environment and affect how they evaluate the consequences of choices, which in turn influences their environmental preferences and behaviours. As values influence the goals that people strive to achieve, they affect environmental behaviour mainly in an indirect manner via specific beliefs and norms (Brosch and Sander, 2015). For example, individuals with strong biospheric values are more likely to consider environmental consequences when making choices, while individuals guided by hedonic values are more likely to make choices to increase pleasure and good feelings (Steg et al., 2014). In this research, we also consider environmental values as a factor influencing environmental decision-making. In other words, when environmental problems threaten individual values, people will care about environmental problems. Consumers’ intention towards agritourism depends on their own environmental values and individual norms, and the intention derived from people’s internal initiative has long-term significance for environmental protection (Pan & Wang, 2018). Thus, we consider environmental values to be a vital factor influencing environmental decision-making.

Agritourism behaviour is a type of pro-environmental behaviour. Existing studies have demonstrated the value of the TPB and VBN theory because they emphasize the subjective initiative undergirding behaviour (Choi et al., 2015; Whitley et al., 2018). The TPB was formally proposed by Ajzen in 1991, launching research on pro-environmental behaviour from the perspective of social psychology. The theory asserts that attitudes, subjective norms, and perceived behavioural control affect behavioural intentions, which, together, shape actual behaviour. Stern et al. (1999) proposed a theoretical framework that incorporates the norm-activation model (NAM) (Schwartz, 1977), value-basis theory (Stern, 1999) and the New Environmental Paradigm (NEP; Dunlap et al., 2000) to create value-belief-norm (VBN) theory in relation to environmentalism. It is widely accepted that values promote environmental behaviour through beliefs (Bamberg et al., 2007). Both the TPB and VBN theory have limitations and shortcomings. The TPB is insufficient in predicting the impact of social norms on personal norms and lacks an assessment of differing motives in relation to environmental behaviour, while VBN theory ignores important factors affecting individual behaviour such as attitudes (Bamberg et al., 2007). Therefore, this paper combines the TPB and VBN theory to advance a theoretical model that includes environmental values, attitudes, and behavioural intentions. The selection of variables for environmental values comes from VBN theory.

### Environmental Values and Consumer Intentions

Agritourism is a complex activity that contributes to human and environmental health, as well as the health of rural settlements, which can support the most wanted desideratum, the sustainability of the environment (Ciolac et al., 2019). At the same time, the perspective that people are part of nature also contributes to their intentions towards agritourism. To study the specific relationships between environmental values and consumer intentions, this article incorporates two main aspects of environmental values, i.e., environmental benefits and the human-nature coordination concept.

Environmental benefits refer to the benefits that various functions of the environment bring to people in the process of people using natural resources (Barbieri et al., 2016). On the one hand, natural environments are a source of physical materials, which can be transported out of the environment and used in various ways (Sutton, 2017). These materials may be of value when directly consumed or used to manufacture products that people value. On the other hand, beautiful scenery also provides people with comfortable psychological enjoyment, which is also a kind of environmental benefit that is difficult to measure in monetary terms (Barbieri et al., 2016). Some empirical studies show that the top-ranking motivations for participation and interest in agritourism are environmental benefits (Jolly and Reynolds, 2005). Some scholars have found that based on the growing concern in the United States about environment-related issues, such as the protection of natural resources and habitat conservation, environmental benefits are particularly important for agritourism development (Barbieri et al., 2016).

Human-nature coordination is a concept that indicates when a person considers humans and nature to be equal, mutually beneficial, and harmonious. According to Liu et al. (2020), the human orientation towards nature includes the worship of nature, the conquest of nature, and conformity to nature. Humans and the natural environment are inseparable and interdependent, and thus, they greatly influence each other. Some empirical studies have found that the human-nature coordination concept significantly, positively, and directly influences people’s attitudes towards tourism and nature conservation (Fraj and Martinez, 2006). These studies also reveal that tourists with the human-nature coordination concept are more likely to support tourism development and nature conservation than those who hold a concept that prioritizes humans (Cheng et al., 2011). Based on these factors, the following hypotheses are proposed:

**H1.** Environmental benefits have a direct positive effect on consumer intentions.

**H2.** The human-nature coordination concept has a direct positive effect on consumer intentions.

### Environmental Values and Attitudes, Subjective Norms, and Perceived Behavioural Control

#### Environmental Values and Attitudes

Attitude refers to an individual’s stable psychological tendency towards a particular behaviour (Ajzen, 1991). If a person has a positive attitude towards a particular behaviour, he or she will be more likely to engage in that behaviour (Ajzen, 1991). There are many factors that may influence people’s attitudes towards agritourism. Environmental values, which include environmental benefits and the human-nature coordination concept, play critical roles in people’s attitudes towards agritourism. Some empirical studies show that people who value environmental benefits more have more positive attitudes towards agritourism (Barbieri et al., 2016; Nguyen et al., 2018). In addition, other studies have found that possessing the human-nature coordination concept has a positive influence on people’s supportive attitudes towards agritourism and nature conservation (Cheng et al., 2011). Additionally, positive attitudes towards agritourism have a positive association with the intention to actively participate in agritourism (Nickerson et al., 2001). On this basis, the following hypotheses are proposed:

**H3.** Attitudes are an intermediary between environmental benefits and consumer intentions.

**H4.** Attitudes are an intermediary between the human-nature coordination concept and consumer intentions.

#### Environmental Values and Subjective Norms

Subjective norms refer to the belief that an important person or group of people will approve and support a particular behaviour (Ajzen, 1991; Liu et al., 2020). Subjective norms are determined by the perceived social pressure from others for an individual to behave in a certain manner and the individual’s motivation to comply with those people’s views. In short, subjective norms refer to an individual’s estimate of the social pressure of his or her participation (or non-participation) in the behaviour under consideration (Ajzen, 1991). According to Li et al. (2019), “the role of social norms is related to behavior that is more or less open to others.” In the era of digital communication, people are more willing to ask others for advice before travelling, and they often share their tourism experiences on social media. As they have more opportunities to access other people’s thoughts and behaviours in regard to environmental benefits and the human-nature coordination concept, their subjective norms are impacted by those who are important to them. On this basis, the following hypotheses are proposed:

**H5.** Subjective norms are an intermediary between environmental benefits and consumer intentions.

**H6.** Subjective norms are an intermediary between the human-nature coordination concept and consumer intentions.

#### Environmental Values and Perceived Behavioural Control

Perceived behavioural control refers to an individual’s “perception of the ease or difficulty of performing the behavior of interest” (Ajzen, 1991:183). It reflects an individual’s perceptions guided by experience or second-hand information, including both internal control factors, such as personal abilities and emotions, and external control factors, such as opportunities, obstacles and information (Ajzen, 1991). Sometimes, perceived behavioural control and actual behavioural control diverge significantly. To the extent that perceived behavioural control is veridical, it can serve as a proxy for actual behavioural control and contribute to predicting the behaviour in question (Yang-Wallentin et al., 2004). In agritourism, people’s perceived behavioural control is generally impacted by time, money, work, and other situations. Using data from a survey in China, some empirical studies have revealed that perceived behavioural control has a positive and significant effect on sustainable consumption behaviours (Wang et al., 2014; Liu et al., 2020). From the perspective of environmental values, agritourism is a kind of sustainable consumption behaviour; thus, it should also apply to the above conclusion. To test the conclusion, it is necessary to further address perceived behavioural control. Accordingly, the following hypotheses are proposed:

**H7.** Perceived behavioural control is an intermediary between environmental benefits and consumer intentions.

**H8.** Perceived behavioural control is an intermediary between the human-nature coordination concept and consumer intentions.

### Attitudes, Subjective Norms, Perceived Behavioural Control and Consumer Intentions

In the current formulation of the theory, favourable attitudes and supportive subjective norms provide the motivation to engage in a behaviour; however, a concrete intention to do so is formed only when the perceived control over the behaviour is sufficiently strong (Ajzen, 2020). Ajzen assumed that behavioural intentions are a motivational factor that affects behaviour, indicating that individuals are willing to accept the difficulty and effort involved in engaging in a behaviour (Ajzen, 1991; Fisbein & Ajzen, 1975). Existing research on the TPB demonstrates that attitudes, subjective norms, and perceived behavioural control are three significant variables that positively correlate with behavioural intentions (Madden et al., 1992). Applying TPB to consumer intentions towards agritourism, the more positive people’s attitudes towards this behaviour are, the more support they have from family and friends, and the more they can control their behaviour, the more intentions they will have to participate in agritourism. On this basis, the following hypotheses are proposed:

**H9.** Attitudes and consumer intentions have a positive correlation.

**H10.** Subjective norms and consumer intentions have a positive correlation.

**H11.** Perceived behavioural control and consumer intentions have a positive correlation.

**Fig. 1:**
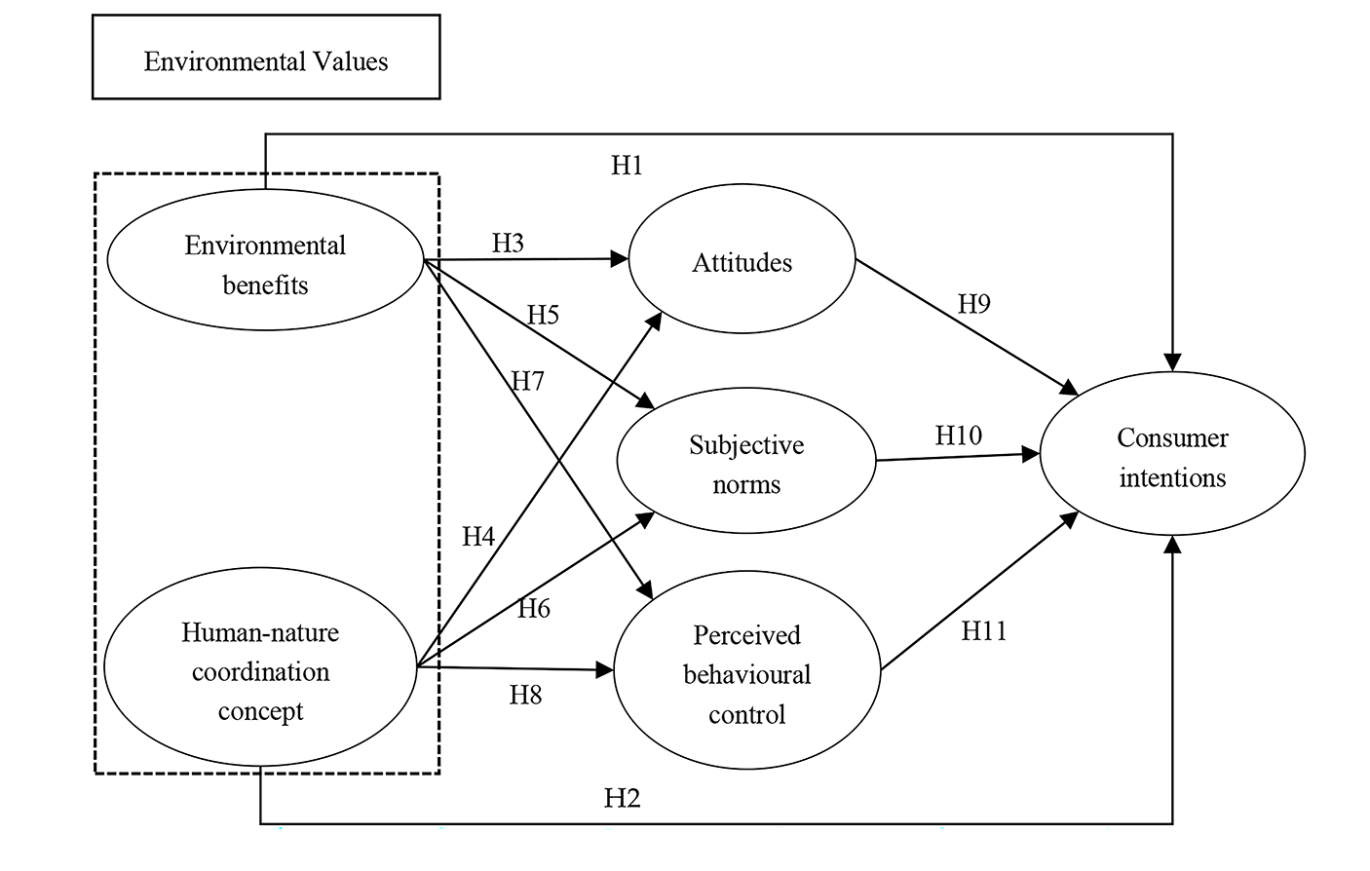
Internal mechanism model of consumer intentions towards agritourism

Based on the above correlations, we create a framework for exploring the mechanisms between consumer intentions towards agritourism, expanding the TPB model. This framework connects environmental values to behavioural attitudes, subjective norms, and perceived behavioural control. These three variables of the TPB impact consumer intentions towards agritourism. First, this study investigates the effects of environmental values on behavioural attitudes, subjective norms, perceived behavioural control, and consumer intentions. Second, it examines the effects of behavioural attitudes, subjective norms, and perceived behavioural control on consumer intentions. Third, it studies the mediating effects of behavioural attitudes, subjective norms, and perceived behavioural control on the relationship between environmental values and consumer intentions towards agritourism. Figure 1 presents the research framework.

## Research Methods

### Scale Design

The survey was designed based on the relevant previous international literature on agritourism to select a suitable scale for the study. It mainly draws from Han et al. (2010), Chen and Tsai (2007), Bruce Traill et al. (2006), Lam and Hsu (2004), Fraj et al. (2006), and Cheng et al. (2011). The survey used in this study consisted of three parts. The first part collected basic information, such as the respondents’ gender, age, region, profession, and income. The second part measured the degree of participation in agritourism. The third part measured subjective variables, including variables related to the TPB (attitudes, subjective norms, perceived behavioural control, behavioural intentions, and perceived moral obligation), consumer intentions, and environmental risks and benefits (environmental risks, environmental benefits, an environmentally friendly lifestyle, and the human-nature coordination concept). The three parts contained a total of 39 items. The measurement contents of the third part are shown in Table 1. To help the respondents better understand the significance of the research and its background, the survey began with a brief introduction to agritourism.

**Table 1:** Variable Design

Latent Variables	Measurement Item	Sources
Environmental Benefits (EB)	EB1: I believe that agritourism will bring benefits to the environment	(Bruce Traill et al., 2006; Fraj and Martinez, 2006; Cheng et al., 2011)
	EB2: I think the natural environment will be protected by agritourism	
Human-Nature Coordination Concept (HN)	HN1: Humans depend on the natural environment for survival	(Bruce Traill et al., 2006; Fraj and Martinez, 2006; Cheng et al., 2011)
	HN2: Environmental protection is important for our future generations	
	HN3: Humans are part of nature	
Attitudes (ATT)	ATT1: It is good for me to engage in agritourism in my spare time	(Han et al., 2010)
	ATT2: It is valuable for me to engage in agritourism in my spare time	
	ATT3: Engaging in agritourism can bring me happiness	
	ATT4: It makes sense for me to engage in agritourism in my spare time	
	ATT5: It is advisable for me to engage in agritourism in my spare time	
	ATT6: I enjoy spending my spare time on agritourism	
	ATT7: I have a positive attitude towards engaging in agritourism in my spare time	
Subjective Norms (SN)	SN8: Those who are important to me support me in engaging in agritourism in my spare time	(Han et al., 2010)
	SN9: Those who are important to me expect me to engage in agritourism in my spare time	
	SN10: Those whose views I respect would like to see me engage in agritourism	
Perceived Behavioural Control (PBC)	PBC11: It is up to me to engage in agritourism in my spare time	(Han et al., 2010)
	PBC12: I can engage in agritourism if I want to	
	PBC13: I have the time and opportunity to engage in agritourism	
Consumer Intentions (INT)	INT1: I’d like to undertake agritourism	(Bruce Traill et al., 2006; Fraj and Martinez, 2006; Cheng et al., 2011)
	INT 2: I’d like to purchase/repurchase products related to agritourism	
	INT 3: I’d like to engage in agritourism with my family	

### Data Collection

Establishing Beijing as the centre, we connect Hebei and Tianjin to form a research system. Geographically, the three regions of Beijing, Tianjin, and Hebei are close neighbours known as the Jingjinji Metropolitan Region. It is the National Capital Region of China, the Capital Economic Zone, and the largest urbanized megalopolis region in North China. It includes an economic region surrounding the municipalities of Beijing and Tianjin along the coast of the Bohai Sea. Agritourism has become an ideal choice for citizens to escape the pressure of urban life at a lower cost in their spare time. In addition, with a developed economy and a large population, Beijing has a large demand for agritourism, while Tianjin and Hebei are rich in agritourism resources within their territories (Lin, 2005; Yuan-yuan, 2009).

The survey and data collection process of this study was conducted by sending online questionnaires through Sojump, a professional online questionnaire survey platform. Respondents completed the questionnaire through a computer or mobile phone to provide detailed feedback on agritourism. The surveys were coded to match each city to facilitate statistical entry. Important measurement items were set as required answers to prevent missing data. Several reasonable logical correlations between each measurement item were set to ensure efficiency. To ensure the validity of the data collection process, we first issued 30 questionnaires in Beijing, Tianjin, and Hebei Province for a preliminary investigation to ensure the validity of the questionnaire.

The formal questionnaire survey was conducted from June 2018 to July 2018. The survey was closed after responses had not been received for several consecutive days. We applied multi-stage, random stratified sampling (Lhoitka and Ringe, 2012) and obtained 640 completed surveys out of 800 total that were distributed. This amount represents an effective response rate of 80%, which is consistent with Byrne’s method (Byrne, 2013) regarding sample selection requirements. Invalid questionnaires were eliminated by manual identification, including 34 responses whose answer time was less than 180 s, and 67 responses that were in patterns such as 5555, 6666, or 4444. The composition and distribution of the sample are shown in Table 2.

Through descriptive statistical analysis of the sample, we found that 69.2% of the respondents have participated in agritourism. In terms of the gender distribution, the number of female respondents was higher than that of male respondents, at 61.4% and 38.6%, respectively. In terms of the age distribution, 75.5% of the respondents were between 19 and 35 years old, of whom 40% were between 19 and 25 years old and 35.5% were between 25 and 35 years old. The specific information is shown in Table 2.

**Table 2:** Descriptive statistics of the sample

Variable Characteristics	Classification Standards	Frequency	Percentage (%)
Gender	Male	247	38.6
	Female	393	61.4
Age	18 and below	38	5.9
	19 ~ 25	256	40
	26 ~ 35	227	35.5
	36 ~ 50	98	15.3
	51 ~ 65	19	3.0
	66 and above	2	0.3
Marital Status	Married	302	47.2
	Single	338	52.8
Ever Participated in Agritourism	Yes	443	69.2
	No	197	30.8
City of Residence	Beijing	221	34.5
	Hebei	215	33.6
	Tianjin	204	31.9
Number of Times of Participation in Agritourism in the Last Year	0	197	30.8
	1	153	23.9
	2	166	25.9
	3	78	12.2
	4	28	4.4
	5	8	1.3
	6	4	0.6
	7 and above	6	0.9
Permanent Family Member Status	Three Generations	93	14.5
	With Spouse and Children	228	35.6
	With Parents	222	34.7
	With Children	7	1.1
	Live Alone	90	14.1
Total		640	100

### Statistical Analysis

During this study, the internal consistency of the data was analysed using SPSS 20.0. Table 3 describes the conclusions of the reliability analysis of the questionnaire. The results indicate that for the six latent variables, Cronbach’s alpha was greater than 0.7. From Table 3, most of the alpha values were above 0.7, and for the two special variables, environmental benefits and perceived behavioural control, the values were above 0.6 and close to 0.7. Therefore, we can conclude that the research variables have high internal consistency and reliability and that the survey data are reliable.

**Table 3:** Reliability test of the latent variables

Latent Variables	Number of Observed Variables	Cronbach’s Alpha
Environmental Benefit (EB)	2	0.659
Human-Nature Coordination Concept (HN)	3	0.878
Attitudes (ATT)	4	0.875
Subjective Norms (SN)	3	0.771
Perceived Behavioural Control (PBC)	3	0.676
Customer Intentions (INT)	3	0.724

Starting from the six dimensions of agritourism, this research adopts SEM and discusses the complicated relationships among the six dimensions, including environmental values (environmental benefits and the human-nature coordination concept), attitudes, subjective norms, perceived behavioural control, and consumer intentions. The SEM includes two sets of theoretical models. The first is the structural model, which is used to define both the potential independent variables (i.e., environmental benefits, the human-nature coordination concept, attitudes) and the potential linear relationship between the variables (i.e., subjective norms, perceived behavioural control). The second is the measurement model, which defines the linear relationships between the latent and observed variables.

## SEM Check

**Fig. 2:**
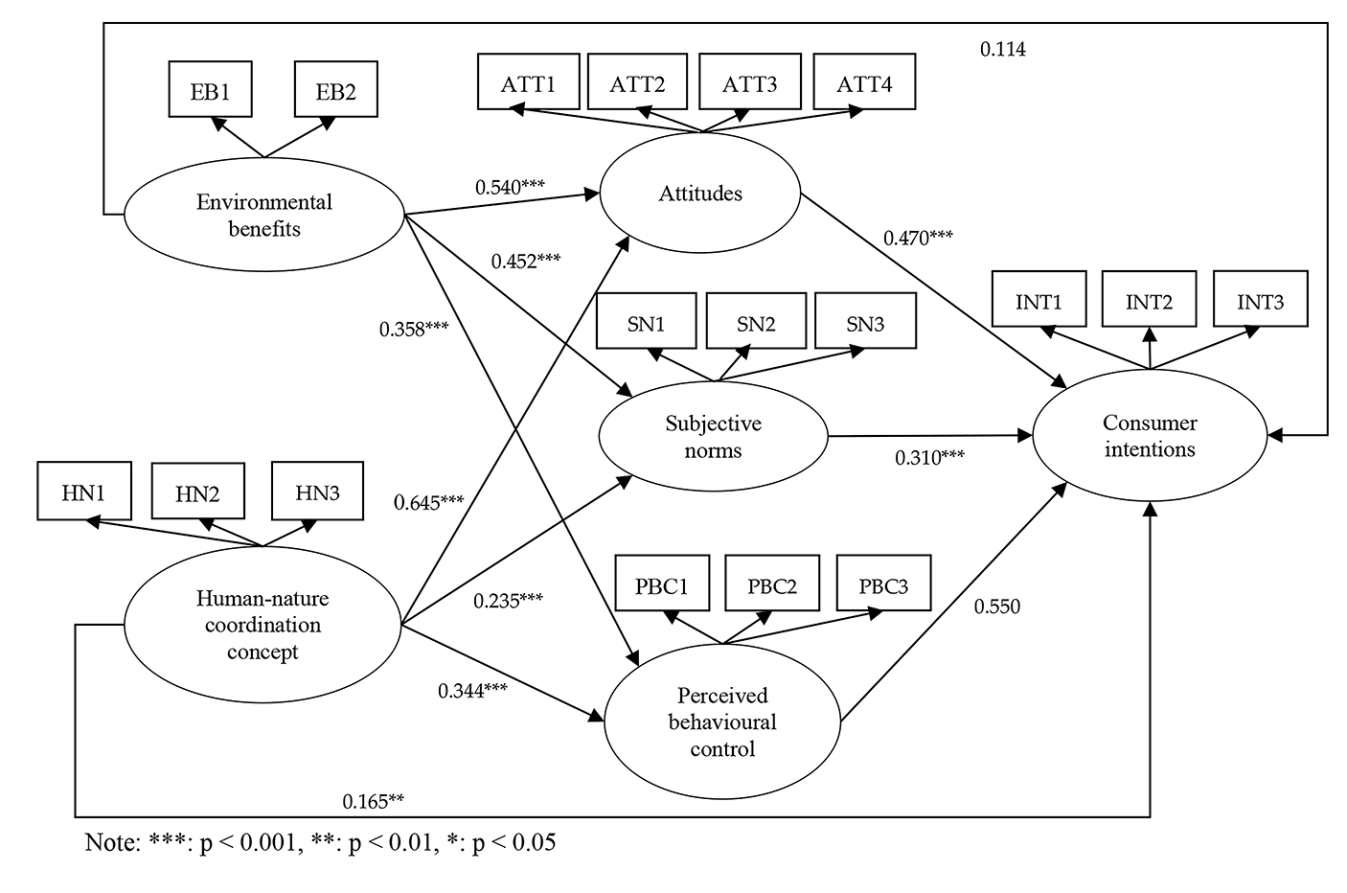
Path graph of the structural equation model (SEM). Note: ***: p < 0.001, **: p < 0.01, *: p < 0.05

Based on the reliability and validity of the survey, this study used the SPSS 20.0 and AMOS 17.0 software analysis tools to establish the structural equation model to analyse the relationship between these six variables. AMOS 22 17.0 software was used to establish the SEM path, as shown in the conceptual model in Fig. 2. Any path that did not pass the inspection was deleted, and the model was adjusted accordingly. The revised conceptual model and its solution path coefficients are shown in Fig. 2. The results demonstrate that the SEM confirmed Hypotheses H2, H3, H4, H5, H6, H7, H8, H9, and H10, while H1 and H11 were not confirmed.

### Model Goodness-of-Fit Test

As shown in Table 4, the SEM goodness-of-fit test was conducted after the model was adjusted. Table 4 shows that the fit index card and degrees of freedom are 3.503; the GFI and NFI values are 0.930 and 0.914, respectively; the IFI and CFI values are 0.937 and 0.937, respectively; and the RMSEA is 0.063, which is less than 0.08, thus confirming that the model is based upon a representative sample. The test results of the SEM check are presented in Table 5.

**Table 4:** Goodness-of-fit test results

	X^2^	Df	X^2^/df	GFI	NFI	IFI	CFI
Standard Effect	430.844	123	< 3 3.503	> 0.9 0.930	> 0.9 0.914	> 0.9 0.937	> 0.9 0.937

**Table 5:** SEM check

H		Path		Estimate	SE	CR	P	Pass or Not
H1	Consumer intentions	←	Environmental benefits	0.114	0.076	1.506	0.132	no
H2	Consumer intentions	←	Human-nature coordination concept	0.165***	0.055	2.969	0.003	yes
H3	Attitudes	←	Environmental benefits	0.540***	0.075	7.232	0.001	yes
H4	Attitudes	←	Human-nature coordination concept	0.645***	0.052	12.321	0.001	yes
H5	Subjective norms	←	Environmental benefits	0.452***	0.069	6.555	0.001	yes
H6	Subjective norms	←	Human-nature coordination concept	0.235***	0.046	5.170	0.001	yes
H7	Perceived behavioural control	←	Environmental benefits	0.358***	0.072	4.954	0.001	yes
H8	Perceived behavioural control	←	Human-nature coordination concept	0.344***	0.053	6.446	0.001	yes
H9	Consumer intentions	←	Attitudes	0.470***	0.048	9.758	0.001	yes
H10	Consumer intentions	←	Subjective norms	0.310***	0.054	5.787	0.001	yes
H11	Consumer intentions	←	Perceived behavioural control	0.055	0.049	1.107	0.268	no

Note: ***: p < 0.001, **: p < 0.01, *: p < 0.05

### Mediating Effect Analysis

The bootstrap intermediate test is a highly effective and widely accepted method of examining mediating effects (Li et al., 2019). To further explore the mechanisms of the impact of environmental values on consumer intentions towards agritourism, this paper applies bootstrapping to further analyse the mediating effect of three variables: attitudes, subjective norms, and perceived behavioural control.

**Table 6:** The results of the bootstrap mediating effect test

Variables	Point Estimate	Boot SE	Bootstrapping	
			Bias-corrected (95% CI)	Percentile (95% CI)
			Lower Upper	Lower Upper
Environmental Benefits - Intentions (Indirect)	0.413***	0.244	0.229 1.174	0.211 1.085
Human-Nature Coordination Concept - Intentions (Indirect)	0.370***	0.103	0.156 0.519	0.073 0.487

Note: ***: p < 0.001, **: p < 0.01, *: p < 0.05

As shown in Table 6, the confidence intervals of the total indirect effect of the environmental benefits-intentions relationship are (0.229, 1.174) and (0.211, 1.085), which do not include 0. These results indicate that the total indirect effect of environmental benefits and intentions exists. Moreover, the confidence intervals of the total indirect effect of –the human-nature coordination concept-intentions relationship are (0.156, 0.519) and (0.073, 0.487), which do not include 0. These results indicate that the total indirect effect of the human-nature coordination concept and intentions also exists.

**Table 7:** The results of the bootstrap mediating effect test of the three variables

Variables	Estimate	S.E	Standardized Total Effects	Mackinnon PRODCLIN 2 95% CI	
				Lower Upper	
Environmental Benefits – Attitudes Attitudes – Intentions	0.540*** 0.470***	0.075 0.048	0.424	0.161	0.364
Environmental Benefits – Subjective Norms Subjective Norms – Intentions	0.452*** 0.310***	0.069 0.054	0.424	0.074	0.225
Environmental Benefits – Perceived Behavioural Control Perceived Behavioural Control – Intentions	0.358*** 0.055	0.072 0.049	0.424	-0.012	0.065
Human-Nature Coordination Concept – Attitude Attitudes – Intentions	0.605*** 0.470***	0.049 0.048	0.515	0.202	0.379
Human-Nature Coordination Concept – Subjective Norms Subjective Norms – Intentions	0.221*** 0.310***	0.043 0.054	0.515	0.032	0.117
Human-Nature Coordination Concept – Perceived Behavioural Control Perceived Behavioural Control – Intentions	0.322*** 0.055	0.050 0.049	0.515	-0.011	0.057

Note: ***: p < 0.001, **: p < 0.01, *: p < 0.05

To further explore the specific effects of the three mediating variables in our model, we further analysed their mediating effects and compared their results, as shown in Table 7. Based on these results, we conclude that, except for two variable relationships, the mediating effects of all the other variables exist, as their confidence intervals do not contain 0. The two variable relationships whose confidence intervals contained 0 were the environmental benefits-perceived behavioural control-intentions relationship and the human-nature coordination concept-perceived behavioural control-intentions relationship, of which the confidence intervals of their mediating effects are (-0.012, 0.065) and (-0.011, 0.057), respectively. Since these two confidence intervals contain 0, their mediating effects do not exist.

**Table 8:** The results of the direct effect test

Variables	Point Estimate	Boot SE	Bootstrapping	
			Bias-corrected (95% CI)	Percentile (95% CI)
			Lower Upper	Lower Upper
Environmental Benefits - Intentions (Direct)	0.114	0.401	-0.130 0.847	-0.053 1.376
Human-Nature Coordination Concept - Intentions (Direct)	0.154***	0.077	0.017 0.297	0.039 0.342

Note: ***: p < 0.001, **: p < 0.01, *: p < 0.05

As shown in Table 8, the confidence intervals of the direct effects in the relationship between environmental benefits and intentions are (-0.130, 0.847) and (-0.053, 1.376), respectively, and both intervals include 0. The direct effect of the relationship between environmental benefits and intentions does not exist, so their mediating effect involves full mediation. However, the confidence intervals of the direct effects in the relationship between the human-nature coordination concept and intentions are (0.017, 0.297) and (0.039, 0.342), respectively, and do not include 0. Therefore, the relationship between the human-nature coordination concept and intentions involves partial mediation.

## Discussion

In the existing literature, few studies reveal the mediating mechanism between environmental values and consumer intentions to participate in agritourism. Based on past studies on environmental values and consumer intentions towards agritourism, this study applies the TPB to the psychological path factors that impact consumer intentions concerning agritourism. Furthermore, it introduces environmental benefits and the human-nature coordination concept, provides conclusions with practical significance, and proposes an extended TPB model. In this study, we found that extending the traditional TPB model augments our understanding of the relationship between environmental values and consumer intentions towards agritourism.

In terms of attitudes, based on the results verifying H9, people’s attitudes towards agritourism are positively related to their intentions in relation to agritourism. In other words, the intentions of people with positive attitudes towards agritourism are stronger. In terms of subjective norms, this study reveals that there is a significant positive correlation between people’s subjective norms towards agritourism and their intentions. This finding means that the support that people receive from important family members and friends positively correlates with their intentions to participate in agritourism. In terms of perceived behavioural control, the results show that the correlation between people’s perceived behavioural control and their intentions towards agritourism is not significant, which is different from the findings of Panwanitdumrong and Chen (2021) and Gkargkavouzi et al. (2019). Perceived behavioural control is affected by many factors, such as perceptual promotion factors and people’s perception of their own ability, resources and opportunities. The nonsignificant relationship shows that, currently, people’s relevant conditions are not enough for them to consume agritourism.

While the results of the above variables extend past research on the basic variables of the TPB, this study extends the analysis by including the relationship between two environmental value variables (environmental benefits and the human-nature coordination concept) and people’s intentions towards agritourism. The results show that environmental benefits have a smaller effect coefficient on consumer intentions towards agritourism. Based on the results of H2, the human-nature coordination concept has a direct and positive correlation with consumer intentions towards agritourism.

However, it is too early to draw the conclusion that environmental benefits have little influence on consumer intentions towards agritourism. The results verifying H3, H4, H5, and H6 show that both environmental benefits and the human-nature coordination concept can indirectly influence consumer intentions towards agritourism by influencing attitudes and subjective norms. Therefore, environmental values influence consumer intentions towards agritourism. In this model, environmental benefits influence intentions towards agritourism through full mediation, while the human-nature coordination concept influences intentions towards agritourism through partial mediation.

The analysis reveals that in the Beijing-Tianjin-Hebei region, a fast-paced and high-pressure urban area, environmental values have a significant positive mediating effect on consumer intentions towards agritourism. Based on the analysis of the mechanisms of the factors that influence consumer intentions towards agritourism, there are several implications for policy-makers and programme implementation.

First, as shown in the empirical tests, the relationship between environmental benefits and consumer intentions towards agritourism involves full mediation. Environmental benefits positively influence consumer intentions towards agritourism through people’s attitudes. Therefore, policy-makers should positively advocate the environmental benefits of agritourism, which will positively affect people’s attitudes and thus increase their intentions towards agritourism. Second, as shown in the empirical tests, the relationship between the human-nature coordination concept and consumer intentions towards agritourism involves partial mediation, which means that the human-nature coordination concept has both direct and indirect impacts on consumer intentions. Therefore, policy-makers should actively promote the human-nature coordination concept to improve subjective norms through social impact, thus increasing intentions. Third, while promoting agritourism, policy-makers should also improve education regarding people’s environmental protection awareness. Improving the characteristics and innovations surrounding tourism projects is an effective way to enhance the attractiveness of agritourism. For example, adding experiential activities with unique local features and encouraging consumers to participate in designing souvenirs that are related to environmental benefits and the human-nature coordination concept will improve consumers’ experience, thus helping consumers and local businesses establish a stable and long-term relationship.

To strengthen the protection of the ecological environment and culture in agricultural areas, the government should also pay attention to culture with local characteristics, learn from existing ancient towns and villages with rich native characteristics, and use unique customs, the historical background, ecology, and the humanities to create tourism villages with original features. Doing so will not only preserve traditional Chinese culture but also integrate tourism, attracting urban consumers to agritourism and promoting capital flow, which may narrow the gap between urban and rural areas. As one of the “seven major strategies” for beginning a new era of socialism with Chinese characteristics, the implementation of agritourism not only embodies the ideal of the “three rural issues” proposed by Xi Jinping but also aligns with the longing for the good life of farmers (Wang and Zhuo, 2018). The implementation of the strategy for rural revitalization requires the cooperation of the government, enterprises, and the masses. The government needs to formulate a series of preferential policies, increase financial allocations, coordinate, and strengthen supervision. Enterprises, especially agricultural-related enterprises, should actively respond to the call and provide more employment opportunities in rural areas.

## Conclusions

Agritourism strengthens the linkages between tourism and agriculture while fostering sustainability principles (Broccardo et al., 2017). It not only provides urban consumers with the opportunity to enjoy and reinforce the atmosphere of agricultural life but also helps agricultural entrepreneurs increase their income by providing tourism services, creating additional employment, and improving local infrastructure (Tew and Barbieri, 2012). Therefore, this study contributes to socio-psychological research on agritourism behaviour by incorporating VBN theory and the TPB by adding two variables, i.e., environmental benefits and the human-nature coordination concept, to the TPB. Through preliminary research, questionnaire design, and surveys, the impact mechanisms were analysed in depth. The main conclusions are as follows:

First, this study found that research on the influencing factors and the mechanisms behind consumer intentions towards agritourism under the “environmental values-attitudes-behavioural intentions” extended framework has scientific applicability through reliability and validity analysis and hypothesis testing of the model. The study provides new theoretical support for research on the public’s intentions towards agritourism.

Second, the two variables of environmental values have a positive correlation with people’s intentions to participate in agritourism directly or indirectly. Environmental benefits influence intentions towards agritourism through full mediation, while the human-nature coordination concept influences intentions towards agritourism through partial mediation (both direct and indirect influence).

Moreover, attitudes and subjective norms are positive mediators between environmental values and consumer intentions towards agritourism. In contrast, there is no significant mediating effect of perceived behavioural control between environmental benefits and the human-nature coordination concept on intentions, indicating that the public’s perception of their ability or confidence to consume agritourism cannot be improved solely by enhancing environmental values to stimulate intention. This result gives us a meaningful indication that heightened levels of opportunity and resources such as time, money, skills, and others’ cooperation are needed to foster behavioural intentions (Ajzen, 1991). This research developed a systematic framework to test a more comprehensive model, tested several hypotheses with empirical data, and provided practical implications to policy-makers. In the future, the government should pay attention to improving people’s awareness of environmental protection and the human-nature coordination concept through various methods, such as policy guidance and cultural cultivation.

However, the conclusions of this study are preliminary. The limitations of this study include three main issues. First, people’s intentions towards agritourism may also be affected by other factors, such as travel costs and education; thus, the control variables could be stricter. Second, the survey sample could be increased, and the survey scope could be extended. Finally, the data were based on self-reported intentions and behaviours. Therefore, subsequent analyses employing data from larger survey samples and the application of alternative statistical methods to verify the hypotheses are clearly needed.
